# Micro-CT evaluation of frozen and embalmed human cadavers on the effect of root canal preparation on microcrack formation in old dentin

**DOI:** 10.1371/journal.pone.0281124

**Published:** 2023-01-30

**Authors:** Franziska Haupt, Christian Dullin, Marcel Krebs, Ingrid Hettwer-Steeger, Philipp Kanzow, Tina Rödig

**Affiliations:** 1 Department of Preventive Dentistry, Periodontology and Cariology, University Medical Center Göttingen, Göttingen, Germany; 2 Department of Diagnostic and Interventional Radiology, University Medical Center Göttingen, Göttingen, Germany; 3 Department of Pathology, University Medical Center Göttingen, Göttingen, Germany; 4 Center of Anatomy, University of Göttingen, Göttingen, Germany; University of the Pacific - Arthur A Dugoni School of Dentistry, UNITED STATES

## Abstract

The aim of this study was to evaluate the existence of preoperative dentinal defects among differently preserved dentoalveolar bone-blocks (frozen vs. embalmed) and to investigate the effect of varying apical forces (low: <4 N, high: 4–8 N) during root canal preparation on microcrack formation using micro-computed tomography (micro-CT). Thirteen embalmed and seven frozen bone-blocks containing 1–3 single rooted teeth were collected. The teeth were evenly divided into three groups (n = 10): F_Low_ (frozen, <4 N), E_Low_ (embalmed, <4 N), E_High_ (embalmed, 4–8 N). After working length determination all specimens were scanned preoperatively. Root canal preparation was performed using nickel-titanium instruments sizes 25/.06 and 40/.06 (F6 SkyTaper; Komet, Lemgo, Germany). A postoperative scan was performed and image stacks were co-registered. All cross-sectional images were screened to identify the presence of dentinal defects. The results were expressed as the percentage of teeth/slices presenting dentinal defects. The statistical analyses were performed with Kruskal-Wallis-Test and Mann-Whitney-U-Test (α = 5%). Embalmed specimens presented a significantly higher percentage of slices with preoperative microcracks (p<0.05) than frozen specimens. No significant difference between groups was observed regarding the induction of microcracks (p>0.05). Root canal preparation does not induce microcracks in dentoalveolar bone-blocks from donors of old age, irrespective of the preservation method and the apically directed forces.

## Introduction

Vertical root fractures occur with a prevalence of 8.8–29.6%, and are one of the major causes for endodontically treated teeth eventually having to be extracted [1–3]. Throughout the years several etiological theories on vertical root fractures have been discussed, including factors such as the patients´ age [4, 5], anatomical variations of the roots [6], bruxism [7], and tooth substance loss [8], especially in the cervical region [9]. Moreover, endodontic procedures, such as lateral compaction [6] and root canal preparation [9–11], have also been presumed to induce dentinal microcracks. Nevertheless, the cause-effect relationship between the existence of microcracks and the development of VRF is still not completely understood.

The first studies investigating dentinal microcrack formation used a fairly destructive method: after root canal preparation the specimens were sectioned and the dentinal surfaces were subsequently microscopically examined [11–13]. However, this experimental set-up is associated with numerous shortcomings, such as the destructive nature, the limited two-dimensional inspection of only specific root sections and the necessity of a control group. Hence, later studies implemented the use of micro-computed tomography (micro-CT) which allows the longitudinal and three-dimensional assessment of the specimens throughout the experimental procedures [14–20]. This highly accurate and non-destructive technology demonstrated that the causal relationship between root canal preparation and microcrack formation is missing [15, 21]. However, micro-CT studies using extracted teeth reported a significant number of preoperative dentinal defects [14–16, 20, 21]. Conversely, on the basis of an innovative approach by performing a micro-CT analysis on an *in situ* cadaveric model, enabling the maintenance of the attachment apparatus’ viscoelastic properties, the existence of such preoperative defects was questioned [22]. For these studies, frozen dentoalveolar bone-blocks were received from young donors with a mean age of 23 and 31 years, respectively [22, 23].

Another important methodological aspect that must be considered with the microcrack research field is ageing, as fatigue crack growth resistance of human dentin decreases with the patients´ age [24]. In extracted teeth, the prevalence of preoperative microcracks in non-endodontically treated teeth was found to be significantly higher in old than in young patients [17]. Moreover, recent micro-CT studies using dentoalveolar bone-blocks from old donors aged between 60–80 years and between 45–95 years identified in 9.5% respective 50% of the specimens preoperative defects [25, 26].

So far, there are only few studies available combining the *in situ* cadaveric model with micro-CT analysis when investigating the phenomenon of microcrack formation [22, 23, 25, 26]. These studies only included one of the two preserved bone blocks: either frozen or embalmed. However, none of these actually compared the two preservation methods with regard to the formation of dentinal microcracks due to root canal preparation. There are multiple studies evaluating the influence of formalin solution on the mechanical properties of bone [27, 28], concluding that formalin fixation may also have an impact on the mechanical properties of dentin. Nevertheless, the exact influence of formalin solution on the biomechanical behavior of dentin is still unknown.

A recent study, however, demonstrated the visibility of dentinal microcracks in frozen and embalmed dentoalveolar bone-blocks not to be affected by the preservation method [29].

As mentioned before, there is a lack of causal relationship between microcrack formation and root canal preparation [15, 19, 21, 30]. In these studies, experienced operators carried out the root canal preparation procedures. As apically directed forces may vary depending on the operators’ experience [31–33], it can be speculated that inexperienced dentists apply higher forces during instrumentation. This is presumed to have an impact on microcrack formation.

The request for a reduced number of instruments in order to facilitate mechanical root canal preparation has led to the development of new nickel-titanium (NiTi) instruments with improved designs, alloys and kinematic movements resulting in a high cutting efficiency and reduced fracture susceptibility. Nevertheless, sharp instruments actively cut into the dentin which results in less sound root structure being more prone to fracture following root canal preparation. Moreover, instrumentation procedures themselves may cause dentinal microcracks depending on the applied forces [34].

Thus, the aim of this study was three-fold: 1) To evaluate the preservation method’s effect on the existence of preoperative dentinal microcracks, 2) to investigate the biomechanical properties of formalin-fixed dentin in comparison with frozen specimens after root canal preparation, and 3) to investigate the effect of high apical forces applied during root canal preparation on microcrack formation.

The null hypotheses tested were that 1) there are no differences between formalin-fixed and frozen specimens regarding the existence of preoperative microcracks, 2) root canal preparation does not induce dentinal microcracks irrespective of the preservation method and the applied apical forces during preparation.

## Materials and methods

### Sample selection

Dentoalveolar bone-blocks were obtained from the Center of Anatomy, University of Göttingen, Germany. A written informed consent is available and was approved by the Ethics committee of the University Medical Center of Göttingen, Germany (no. 23/3/19). Protocols were implemented in accordance with the Helsinki declaration (2013) and relevant guidelines and regulations (Registration on www.drks.de; ID: DRKS00022127). All donors were aged between 64 to 99 years (mean age 82 years) and signed an informed consent for body donation during lifetime. Inclusion criteria were the presence of intact single-rooted teeth surrounded by alveolar bone and periodontal ligament. Periapical radiographs were taken to confirm the presence of a single root canal and to assess the alveolar bone attachment level. Teeth with previous endodontic treatment and an attachment loss of more than 75% were excluded.

Thirteen mandibular and maxillary bone-blocks immersed in 10% formalin and seven frozen mandibular bone-blocks were collected. Embalmed cadavers were stored for approximately two years in the Center of Anatomy without refreshing the formaldehyde solution prior to this investigation. Frozen specimens were kept at a constant temperature of– 80°C before and after sawing. To fit into the micro-CT sample chambers, all jaws were further sectioned into bone-blocks containing the teeth of interest, surrounding alveolar bone and attached mucogingival tissues using a diamond saw (Exact 300, Exact Advanced Technologies GmbH, Norderstedt, Germany; Diamond blade type 300, 0.1mm D64, Walter Messner GmbH, Oststeinbek, Germany). Finally, the soft tissue was removed using a surgical scalpel blade no. 15 and a periosteal elevator. Depending on the number of residual teeth, each segment contained 1–3 single rooted teeth, resulting in a final count of 30. To attain an overall outline of the canal anatomy, all bone-blocks were pre-scanned with an isotropic resolution of 40 μm using a micro-CT scanner (QuantumFX, Perkin Elmer, Waltham, MA, USA) at 90 kV and 200 μA. CTAn v.1.20.3.0 software (Bruker-microCT, Kontich, Belgium) was employed to calculate preoperative quantitative parameters, such as structure model index (SMI), root canal volume and surface area. The teeth were divided into the following three experimental groups (n = 10) according to all preoperatively assessed parameters (attachment level, SMI, root canal volume, surface area):

1. F_Low_: Frozen specimens. Root canal preparation was performed applying adequate axial forces with peaks up to 4 N.
2. E_Low_: Embalmed specimens. Root canal preparation was performed applying adequate axial forces with peaks up to 4 N.
3. E_High_: Embalmed specimens. Root canal preparation was performed applying high axial forces with maximum loads (peaks) between 4 N and 8 N.

Homogeneity of preoperative parameters among the groups was confirmed statistically using nonparametric analysis of variance (p>0.05, Statistica software v. 13.0, StatSoft, Tulsa, OK, USA).

Frozen specimens were stored at– 80°C. They were slowly defrosted prior to root canal preparation by removing them from the freezer and placing them in tap water at room temperature. Root canals were accessed and pre-flared with a 10 size K-file. Working length (WL) was determined radiographically with a 15 size K-file and adjusted to 1 mm short of the radiographic apex.

Afterwards, each specimen was scanned preoperatively using a micro-CT device (SkyScan 1272; Bruker-microCT, Kontich, Belgium) at a high isotropic resolution of 10 μm at 100 kV and 100 μA with a 180° rotation around the vertical axis, a rotation step of 0.15°, a camera exposure time of 2978 ms, and frame averaging of 3. X-rays were filtered with a 0.11-mm-thick copper filter. Recent studies have shown the moisture content to influence the visibility of dentinal microcracks in extracted teeth, as well as in dentoalveolar bone-blocks on micro-CT images [18, 29]. Therefore, all specimens were kept under dry conditions (room temperature, 21°C; 27% humidity) for 24 hours before scanning to allow the detection of dentinal microcracks [29]. For the scan the specimens were placed on dry foam and the sample holder was tightly closed with a plastic foil to maintain a constant humidity for the entire scan duration of 4.5 hours. Images were reconstructed with NRecon software v.1.7.5.9 (SkyScan 1272; Bruker-microCT) using 20% beam hardening correction, ring artefact correction of 20, and smoothing set to 0.

### Root canal preparation

**Fig 1:**
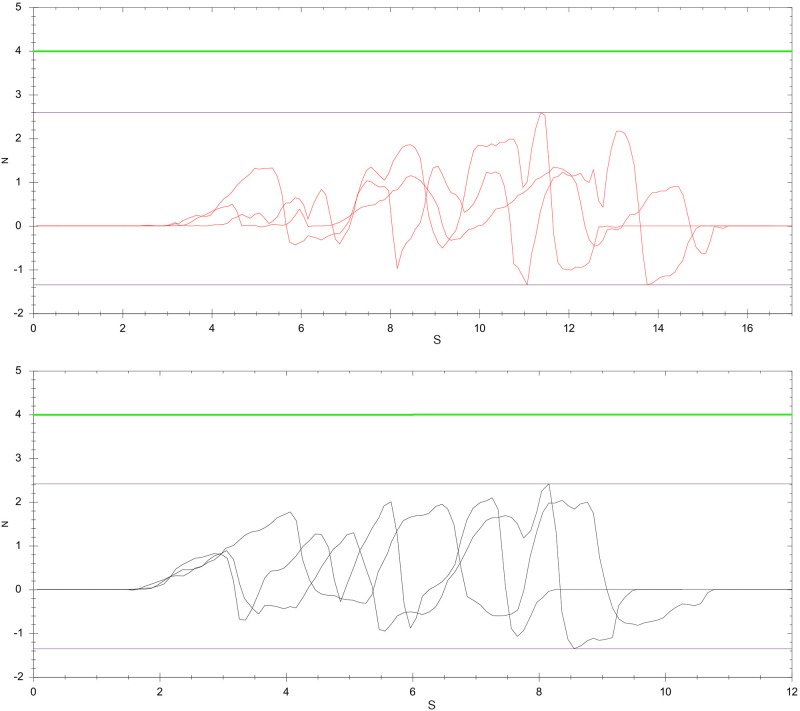
Axial forces applied during root canal preparation of one specimen with peak values less than 4 N. Red: 25/.06; black: 40/.06.

**Fig 2:**
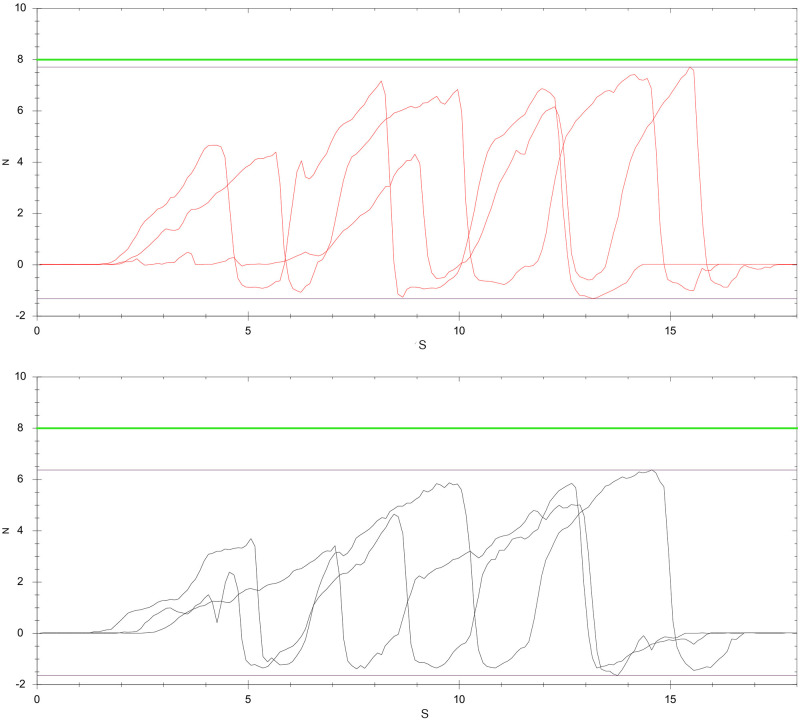
Axial forces applied during root canal preparation of one specimen with peak values between 4 N and 8 N. Red: 25/.06; black: 40/.06.

Root canal preparation was performed using rotary nickel-titanium instruments size 25/.06 followed by size 40/.06 (F6 SkyTaper; Komet, Lemgo, Germany) according to manufacturer’s instructions. Preparation procedures were carried out by a single experienced operator and deemed complete when instruments size 40/.06 taper had reached the WL. Each instrument was used to prepare solely one root canal. After three pecking motions, root canals were irrigated with 3 mL sodium hypochlorite (NaOCl,1%). Altogether each root canal was irrigated with a total of 18 mL NaOCl, followed by a final flush with 5 mL citric acid (10%) and 5 mL NaOCl (1%). According to the experimental group, root canals were prepared with apical forces less than 4 N (Fig 1) or within the range between 4–8 N (Fig 2) which were recorded using a measuring device for mechanical values (USB Sensor Interface Type 9206; DigiVision software v. 2017.1.0.0, Burster, Gernsbach, Germany). Mean maximum forces of 5.2 N ± 1.85, 2.08 N ± 1.1 and 1.66 N ± 0.79 were registered for groups E_High_, E_Low_ and F_Low_, respectively. After root canal preparation, a postoperative micro-CT scan of each specimen was performed using the aforementioned parameters.

### Dentinal microcrack evaluation

The reconstructed pre- and postoperative image stacks were co-registered to obtain an identical positioning of virtual teeth (Data viewer software v. v.1.5.4.0, Bruker-microCT). For the purpose of identifying the presence of dentinal microcracks, two calibrated examiners blinded to the experimental groups screened the cross-sectional images of all roots from the apex to the marginal bone level (N = 33844). This resulted in an acquisition of 760–1492 transverse cross-sections per tooth. All of those postoperative images showing dentinal microcracks were recorded and compared with the corresponding preoperative image to distinguish between preoperative and new microcracks. All images were reassessed after an interval of two weeks. In case of discrepancy, the image was examined together until a consensus was reached [16].

**Fig 3:**
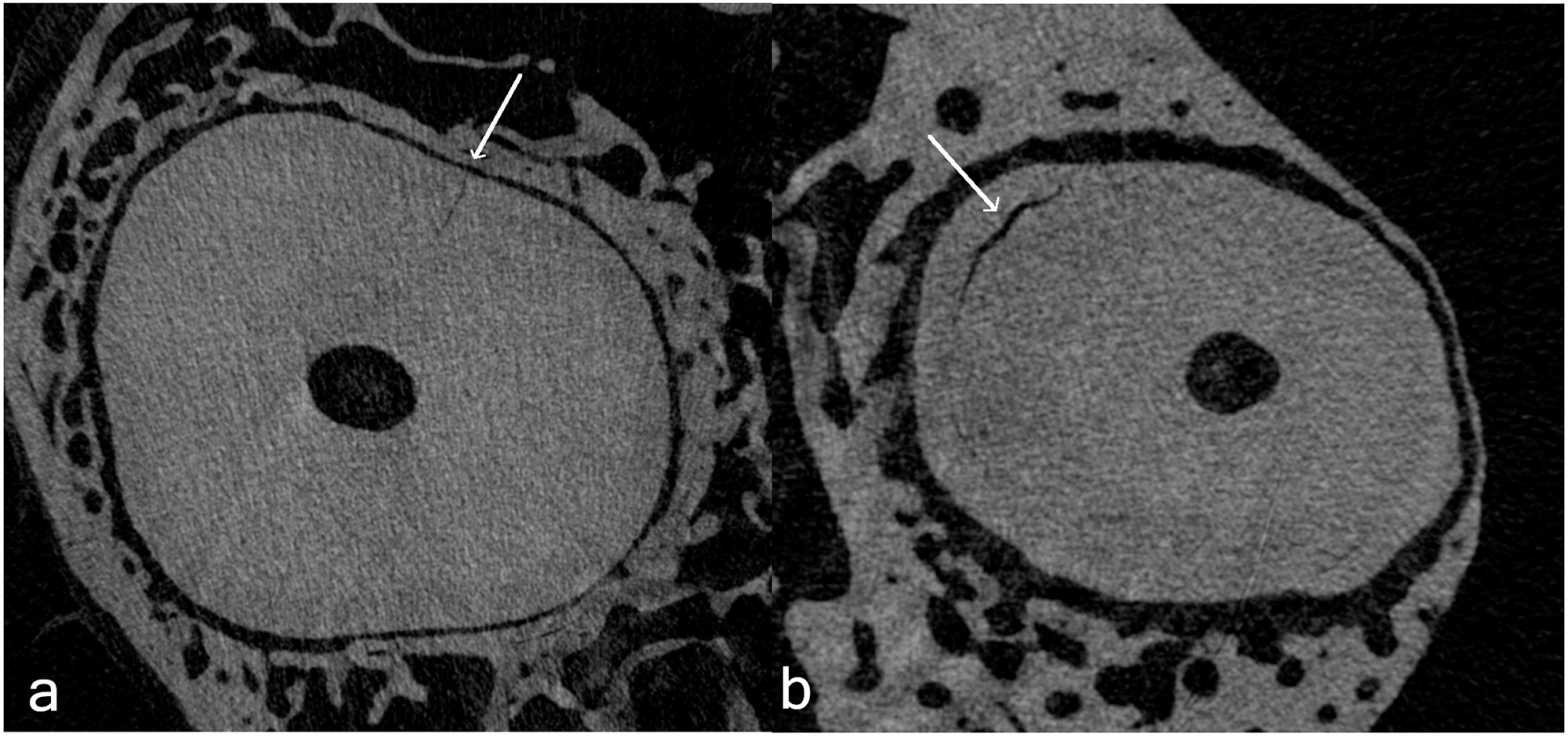
Preoperative microcrack and defect. Representative cross-sectional slices with a preoperative microcrack (a) and defect (b).

In case of preoperative irregularities in dentin, findings were categorized into *microcracks* (cracks directed perpendicular to the root surface, extending from the root canal lumen to the dentin or from the outer root surface into the dentin including complete fractures extending from the root canal all the way to the outer root surface [35, 36] and *defects* (cracks running parallel to the root surface) (Fig 3).

### Statistical analysis

The results were expressed as the percentage of teeth respective slices presenting defects or microcracks. The level of statistical significance was set at α = 5% (IBM SPSS Statistics v. 28.0, IBM, Armonk, NY, USA). The distribution of the data was tested by means of the Shapiro-Wilk-Test, which revealed non-normal distribution for both preservation methods (p_Frozen_ < 0.001; p_Embalmed_ = 0.001). The Kruskal-Wallis-Test was used to compare the presence of preoperative defects between bone-blocks within the same preservation method and to compare experimental groups regarding the induction of new microcracks. Comparison regarding preoperative defects between frozen and embalmed specimens was carried out using the Mann-Whitney-U-Test.

## Results

Regarding the preoperative condition of all bone-blocks within the same preservation group (p_Frozen_ = 0.486, p_Embalmed_ = 0.174), no significant differences were found. The prevalence of preoperative microcracks / defects relating to the preservation method is presented in Table 1. Embalmed specimens presented a significantly higher percentage of slices with preoperative defects than frozen specimens (p = 0.039).

**Table 1:** Absolute and relative frequency of preoperative microcracks/defects for frozen and embalmed specimens at the group-level and percentage of slices with microcracks/defects at the tooth-level.

Preservation method Teeth per group *(n)* Slices per group *(N)*	Absolute and relative frequency of teeth *(n)* and slices *(N)* with microcracks/defects per group		Relative frequency of slices with microcracks/defects per tooth		
	*n/N*	*n*%/*N*%	Median	IQR	Min-max
Frozen	3/459	30/4.06	0.00 ^A^	0.00–10.32	0.00–93.52
*n* = 10, *N* = 11300					
Embalmed	14/5080	70/22.53	12.69 ^B^	0.00–32.11	0.00–24.80
*n* = 20, *N* = 22544					

Different superscript letters indicate statistically significant differences between preservation methods (Mann-Whitney-U-Test). IQR: interquartile range; min-max: minimum and maximum percentage; *n*: number of teeth, *N*: number of slices.

**Fig 4:**
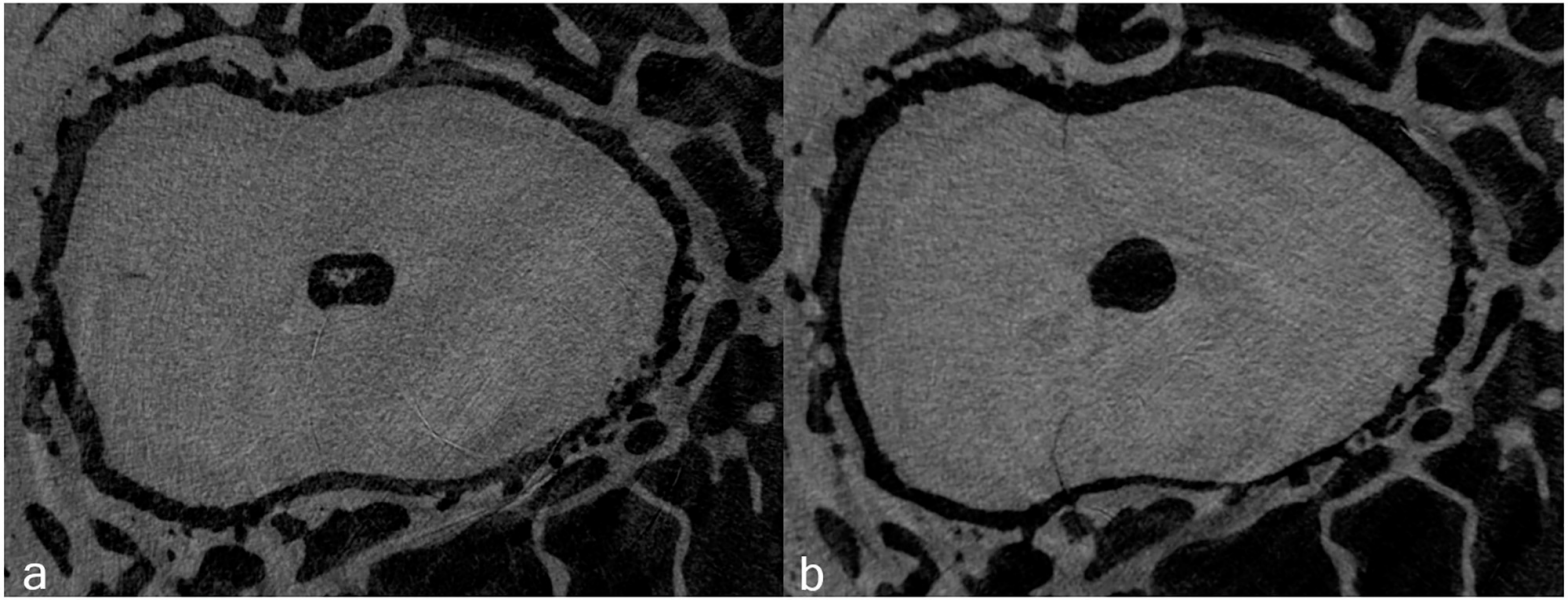
New microcrack after root canal preparation. Representative preoperative (a) and postoperative (b) cross-sectional slices with a new microcrack after root canal preparation.

In group F_Low_ new microcracks were observed in one specimen (1/10). In the groups of embalmed specimens (E_High_ and E_Low_) no new microcracks were detected. The percentage of slices with new microcracks (p = 0.368) did not significantly differ between the experimental groups (Table 2). Newly formed cracks tended to extend from the external root surface into the inner root dentin (Fig 4). All other dentinal microcracks identified in the postoperative scans had already been present in the corresponding preoperative images. Thus, neither the axial force applied during root canal preparation, nor the preservation method showed to have a significant impact on microcrack formation.

**Table 2:** Absolute and relative frequency of new microcracks for each experimental group at the group-level and percentage of slices with new microcracks at the tooth-level.

Experimental group Teeth per group *(n)* Slices per group *(N)*	Absolute and relative frequency of teeth *(n)* and slices *(N)* with new microcracks per group		Relative frequency of slices with new microcracks per tooth		
	*n/N*	*n*%/*N*%	Median	IQR	Min-max
F_Low_	1/366	10/3.24	0.00 ^A^	0.00–0.00	0.00–41.31
*n* = 10, *N* = 11300					
E_Low_	0/0	0/0.00	0.00 ^A^	0.00–0.00	0.00–0.00
*n* = 10, *N* = 10806					
E_High_	0/0	0/0.00	0.00 ^A^	0.00–0.00	0.00–0.00
*n* = 10, *N* = 11738					

Different superscript letters indicate statistically significant differences between experimental groups (Kruskal-Wallis-Test). F_Low_: Frozen specimens, root canal preparation with axial forces less than 4 N; E_Low_: Embalmed specimens, root canal preparation with axial forces less than 4 N; E_High_: Embalmed specimens, root canal preparation with axial forces between 4 N and 8 N; *n*: number of teeth, *N*: number of slices.

## Discussion

Currently, micro-CT imaging is the gold standard tool to analyze the phenomenon of dentinal microcracks. Several studies using micro-CT imaging have demonstrated the existence of dentinal defects which had already been present before performing endodontic procedures [16, 20, 21]. However, these studies included extracted teeth without surrounding bone and periodontal ligament. This tissue may act as a shock absorber [37]. In recent literature, the prevalence of such preoperative microcracks has been doubted for several reasons. First, the extraction of teeth generates forces which are suggested to be involved in microcrack formation [22]. Second, dehydration of specimens during experimental procedures may trigger dentin contraction [38] which potentially results in crack initiation and a reduced fracture strength [39, 40]. Therefore, these preoperative defects have been referred to as *experimental dentinal microcracks* [22]. However, in the present study, preoperative microcracks and defects were observed, despite the use of an *in situ* approach in the form of human cadavers. One main difference to previous studies is the mean age of the body donors. Studies on human bone have shown that, due to aging, collagen undergoes a process of maturation that involves a change in the degree of cross-linking [41]. Non-enzymatic crosslinks form Advanced Glycation End Products (AGEs), which create intra- and interfibrillar cross-linking, resulting in substantial reduction in the strength of bone [42]. In the same way, a high accumulation of AGEs was established in older dentin, which, consequently, becomes harder and more fragile [43]. However, not only the changes in the constitutive behavior of the collagen, but also the increase of mineralization of dentin with age may have an impact on the physical properties and crack growth resistance [24, 44]. Hence, there is a degradation in quality of human dentin with age, apart from the increase in quantity [45].

Whereas the current study included teeth derived from rather old donors with a mean age of 82 years, two previous studies obtained specimens from donors with a mean age of 23 and 31 years [22, 23]. In one of these studies preoperative defects were observed in only one out of sixteen specimens [23]. In contrast, in the present study three out of ten frozen and fourteen out of twenty embalmed specimens presented preoperative microcracks or defects. However, in order to assess solely the influence of the root canal preparation procedures on the formation of microcracks, the preoperative scan was performed after the access cavity preparation. Therefore, the effect of the latter on the existence of preoperative microcracks cannot be entirely excluded. In the current study, the percentage of slices presenting defects displayed statistically significant differences depending on the preservation method. Therefore, the first hypothesis must be rejected. Whether factors, such as the formaldehyde-induced modifications in dentin, the freezing-thawing processes of the frozen specimens, the microstructural changes of dentin during ageing, the marginal alveolar bone loss due to periodontitis, or masticatory forces during lifetime influenced the formation of such defects, can only be speculated.

Despite micro-CT scans being the current gold standard tool for microcrack analysis, there are several limitations associated with this non-destructive technology. Firstly, the detection of dentinal defects is significantly influenced by the isotropic resolution of the micro-CT scan [46]. In the present study, specimens were scanned at an isotropic voxel size of 10 μm which is comparable to previous investigations using a cadaveric model scanned with a resolution of 13.18 μm [22, 23]. Nevertheless, microcracks thinner than 10 μm might not have been detected. Another limitation of micro-CT imaging is the phenomenon of reconstruction image artefacts, which either might be confused with dentinal microcracks or may overlap with an existing microcrack impeding its detection. Nevertheless, performing the sequential analysis of cross-sectional slices throughout the longitudinal axis of the root helps to differentiate between microcracks and artefacts [47]. Moreover, these artefacts mostly appear with extreme contrast differences, for example between dentin and root canal filling materials [48].

Besides the varying scanning and reconstruction parameters among different studies evaluating microcrack formation, which impede comparability of results [14, 16, 17, 49], one of the major drawbacks is the moisture content of the specimen during scanning procedures [47]. It has been shown, that microcracks are blacked out due to excessive moisture condition of dentin, leading to false-negative results [18]. Moreover, the limited visibility of dentinal microcracks in overly wet dentoalveolar bone-blocks was recently demonstrated [29]. Therefore, in the present study, specimens were stored under dry conditions for 24 hours before scanning procedures, enabling the detection of dentinal microcracks. However, the ideal moisture condition of the specimens seems to be a balancing act between the visibility of microcracks and the formation of new defects induced by dehydration [38, 47]. To identify the optimum moisture conditions of specimens in order to detect dentinal microcracks is a major challenge when using micro-CT imaging [18, 29, 47]. Furthermore, the identification of microcracks requires high resolution scans resulting in very long scanning duration up to several hours, in which the moisture content of the sample needs to be kept constant [29]. However, the necessity of drying the specimen prior to the scanning procedure was clearly demonstrated by a recent investigation [29]. Without any doubt, this aspect is one of the major disadvantages when using micro-CT imaging for dentinal microcrack research. To conclude, the differentiation between the detectability of microcracks and their formation due to dehydration remains a major challenge and requires further investigation as long as micro-CT imaging is used in microcrack research.

The second finding of the present study is there being no differences in microcrack formation after root canal preparation when comparing formalin fixed bone-blocks with frozen specimens.

So far, conflicting results in the literature regarding the biomechanical behavior of frozen and embalmed human bone have been reported [27, 50]. However, comparative studies on the effect of root canal preparation on embalmed dentin are scarce [26]. It is well known that formalin solution results in an increased number of cross-linking between collagen fibers and therefore altering the mechanical properties of bone [28, 51]. Moreover, formalin does not only affect the organic tissue, but also the inorganic component of the bone, as calcium-phosphates dissolve in acidic formalin solution. This results in a decreased mineral content and an increased bone porosity [52]. The exact impact of formalin solution on the physical properties of human dentin can only be estimated. Nevertheless, the pH of the solution as well as the temperature during formalin fixation seem to have an effect on the mechanical properties of mineralized tissues [51]. Moreover, it was shown, that several storage solutions may have an influence on the microhardness of dentin over a certain period of time [53]. However, the knowledge regarding the influence of formalin on dentin over time is still limited and its histochemical effect on collagen and elastin, the main components of the organic matrix of dentin, is inconclusive [54, 55], and requires further clarification.

The current study does not demonstrate significant differences between frozen and embalmed dentin regarding microcrack formation after root canal preparation. However, the results are limited regarding the knowledge on the impact of formalin on physical properties of old dentin. Nonetheless, as there is a higher degree of natural crosslinks between collagen fibrils in old dentin due to aging [43], it can be speculated that formalin fixation may have a stronger impact on the mechanical properties of younger dentin. Furthermore, existent natural crosslinks are preserved despite formalin fixation [55]. AGEs, such as pentosidine and pyridinoline, which are present in many connective tissues, cartilage and bone, are not altered or destroyed by formaldehyde solutions [55]. Yet, there is a lack of information about the specific types of AGE molecules incorporated in human dentin.

Whereas numerous previous studies reported an association between root canal preparation and microcrack formation [13, 36, 56], more recent studies questioned the correlation between endodontic procedures and the induction of dentinal defects [19, 22, 23, 30]. In this context, one must mention that the analytic tool in microcrack research has changed considerably over the years. Whereas initial studies assessed microcrack formation by means of a destructive horizontal sectioning method which requires an additional control group [11, 36, 57], recent studies have used micro-CT analysis providing thousands of cross-sectional images. These enable a comparative evaluation of pre- and postoperative image stacks [15, 16, 19, 49], where each specimen serves as its own control. This allows the assessment of the structural condition of dentin in the baseline control images [47]. Moreover, micro-CT analysis in combination with the use of dentoalveolar bone-blocks is considered as the gold standard for dentinal microcrack evaluation, as this model presents the closest to real-life condition by preserving the viscoelastic properties of the periodontal ligament with the surrounding bone [47]. One of the strengths of the current study is the implementation of this novel experimental setup. Nevertheless, the acquisition of such dentoalveolar bone-blocks is time-consuming and challenging. Obtaining suitable samples for the present study, especially fresh-frozen bone-blocks, was very difficult and requires a well working cooperation between different institutions [25]. However, the final sample size of 10 teeth in each group is comparable with recent investigations [25, 46] and complied with previous sample size calculations for such research [23].

The third aim of the present study was to investigate the effect of high apical forces applied during root canal preparation on microcrack formation. Previous studies evaluating physical parameters during root canal preparation assessed forces between 1.26 N and 4.14 N, depending on the preparation technique [34, 58]. Therefore, in the present study, axial loads less than 4 N and within the range between 4–8 N were considered as *adequate* respective *high*. As the applied forces may be influenced by the operator and his experience, one objective of the present study was to assess the impact of high loads applied during preparation on the induction of dentinal microcracks. For root canal preparation, rotary NiTi-instruments sizes 25/.06 and 40/.06 taper were used. This final instrumentation size complied with previous studies including similar tooth types [59, 60]. However, the decision regarding the final preparation size is always a balance between a sufficient root canal debridement and an unnecessary loss of tooth substance. The used instruments exhibit an S-shaped cross-section and, according to the manufacturer´s instructions, only two instruments are required to complete preparation procedures. For the present study this NiTi-system was chosen for two reasons. First, it combines the current request for a reduced number of instruments with an established cross-sectional design. Second, the large continuous taper of 6% facilitates the application of high apically directed loads [33]. It was shown, that the preparation of narrow and constricted root canals is associated with higher axial loads compared to wide root canals [33]. In order to ensure an utmost homogeneous distribution of narrow and wide root canals among the experimental groups, all specimens were scanned preoperatively and anatomical parameters (root canal volumes, surface areas and structure model index) were calculated. After root canal preparation, only one tooth in the group of frozen specimens prepared with adequate axial forces exhibited a new microcrack. Hence, the second hypothesis, that root canal preparation does not induce dentinal microcracks irrespective of the preservation method and the apically directed load, can be confirmed. Nevertheless, these results are limited on the preparation of straight teeth with one single root canal. Further studies on teeth with more complex root canal morphologies are needed to evaluate the effect of high axial forces on microcrack formation, considering aspects, such as different tooth types and severity of root canal curvature.

## Conclusions

Within the limitations of the present study, it can be concluded that the preservation method does not influence the biomechanical response of old root dentin to root canal preparation. Furthermore, inadequate forces during preparation procedures do not seem to induce the formation of dentinal microcracks.

## Supporting information

S1 Dataset (SAV)
